# Determining Public Awareness of the Roles of Dermatologists and Plastic Surgeons: A Survey-Based Study in Saudi Arabia

**DOI:** 10.7759/cureus.89862

**Published:** 2025-08-12

**Authors:** Muna F Alnaim, Saleh M Aldraibi, Omar N Turkistani, Dareen Bajamaan, Hessah A Almojel, Reema H Alghmdi, Shahad F Alotaibi, Mashael D Alotaibi, Munirh O Alomar, Abdulhadi Jfri

**Affiliations:** 1 Emergency Medicine, Al-Jafr General Hospital, Al Ahsa, SAU; 2 Medicine, King Saud Bin Abdulaziz University for Health Sciences, Jeddah, SAU; 3 Medicine, University of Jeddah, Jeddah, SAU; 4 Medicine, Imam Mohammad Ibn Saud Islamic University, Riyadh, SAU; 5 Medicine, Al-Baha University, Al-Baha, SAU; 6 Clinical Laboratory Science, Shaqra University, Shaqra, SAU; 7 Nursing, King Abdullah Bin Abdulaziz University Hospital, Al Ahsa, SAU; 8 Dermatology, King Saud Bin Abdulaziz University for Health Sciences, Jeddah, SAU

**Keywords:** dermatology, health education, patient referral, plastic surgery, public awareness, saudi arabia, social media

## Abstract

Background

Dermatologists and plastic surgeons share overlapping roles in managing skin conditions and performing cosmetic procedures, yet their core responsibilities differ significantly. Dermatologists diagnose and treat skin, hair, nail, and mucous membrane disorders, while plastic surgeons focus on reconstructing and restoring tissue function and appearance. Misunderstandings about these specialties can lead to inappropriate referrals, delayed treatment, and increased healthcare costs. This study aimed to assess public awareness of the roles of dermatologists and plastic surgeons using a scenario-based questionnaire in Saudi Arabia to identify misconceptions and knowledge gaps among the public and inform targeted educational efforts and improve referral accuracy.

Methods

This cross-sectional study was conducted among Saudi residents using a structured, self-administered online survey distributed via social media. The survey included demographic questions and scenario-based assessments of participant understanding regarding the roles of dermatologists and plastic surgeons. Responses were analyzed using IBM SPSS Statistics for Windows, Version 27 (Released 2020; IBM Corp., Armonk, New York, United States), with descriptive statistics used to summarize participant characteristics and awareness levels. Pearson's chi-square test was applied to assess associations between awareness and sociodemographic factors, with statistical significance set at p<0.05.

Results

Of the 882 respondents, 86.2% demonstrated poor awareness regarding the appropriate specialist for common dermatological and cosmetic procedures, while only 13.8% exhibited good awareness. Female participants (14.8%) and single individuals (16.7%) had significantly higher awareness levels (p=0.049 and p=0.023, respectively). Social media was the most commonly reported source of information (79.9%), suggesting its substantial influence on public perceptions. Common misconceptions included the widespread belief that Botox injections are primarily performed by plastic surgeons (55.3%) and a lack of recognition of dermatologists' surgical capabilities, such as in skin cancer treatment.

Conclusion

This study highlights a critical gap in public awareness regarding the roles of dermatologists and plastic surgeons in Saudi Arabia. Targeted educational interventions, particularly through social media, are essential to address these misconceptions, improve patient decision-making, and enhance referral accuracy. Future research should explore the impact of these educational efforts on patient outcomes and healthcare efficiency.

## Introduction

When it comes to skin-related problems and cosmetic operations, the duties of dermatologists and plastic surgeons frequently overlap, leaving patients unsure of whom to visit for particular concerns. Dermatology is a specialized field focused on diagnosing and treating inherited and acquired conditions affecting the skin, hair, nails, and mucous membranes for all age groups. It also involves managing sexually transmitted infections (venereal diseases) using various medical, surgical, and therapeutic methods while addressing the systemic effects of skin disorders and the skin-related signs of systemic diseases [1,2]. 

Plastic surgery, on the other hand, is utilized to repair and reconstruct damaged or missing skin and tissue, with the primary objective of restoring both the function and appearance of these areas to achieve a condition that closely resembles normalcy [3,4].

Since the 2000s, the number of aesthetic and reconstructive surgeries has nearly doubled, according to a newly published article using data from the American Society of Plastic Surgeons members [5]. 

Previous studies have indeed highlighted the challenges patients face in distinguishing between dermatological and plastic surgical services. For example, a study that was conducted in the United States indicated that a significant percentage of patients misidentified the appropriate specialist for their skin-related concerns. This misidentification can lead to patients seeking care from the wrong specialist, which may result in inadequate treatment for their conditions [6]. 

The primary objective of this study is to evaluate the level of awareness and understanding regarding the procedures performed by dermatologists versus plastic surgeons in Saudi Arabia. This was done using a scenario-based questionnaire distributed to the general public. The study aims to identify misconceptions and knowledge gaps, with the goal of informing future educational initiatives and improving the accuracy of the referral process within the healthcare system.

## Materials and methods

This cross-sectional research employed a non-randomized, self-selecting sampling approach targeting residents of the Kingdom of Saudi Arabia. Data collection was conducted through digital platforms, with a structured survey distributed via social media channels, resulting in 880 completed responses. Prior to participation, individuals received comprehensive details regarding the study’s aims and objectives. Eligible participants who provided informed consent were subsequently directed to an electronic questionnaire designed to evaluate their perceptions of dermatological and cosmetic surgical practices.

The questionnaire was built based on previous research [7,8] and aimed to assess the general public’s understanding of the roles and responsibilities of dermatologists and plastic surgeons in the Kingdom of Saudi Arabia. The questionnaire consisted of two sections. The first section sought the demographic details from the respondents, such as their age, gender, place of residence, education level, monthly income, marital status, and the source of their information. In the next section, participants were required to respond to hypothetical clinical scenarios by determining whether each scenario fell under the scope of practice of plastic surgeons, dermatologists, or both or indicating uncertainty ('Don't know'), to assess their understanding of professional roles. Only participants above the age of 18 were included in this survey.

Statistical analysis

Data analysis was performed using IBM SPSS Statistics for Windows, Version 27 (Released 2020; IBM Corp., Armonk, New York, United States). Descriptive statistics, including frequencies and percentages, were calculated to summarize the sociodemographic characteristics of the participants and their responses regarding awareness and perceptions of dermatological and cosmetic procedures. A scoring system was developed to assess participant awareness, assigning one point for each correct answer. Based on these scores, participants were categorized into two groups, reflecting their level of awareness. A cutoff point of 60% correct answers was used to differentiate between poor and good awareness levels. To explore the relationships between awareness levels and various socio-demographic factors (region, age, gender, education, income, and marital status), as well as sources of information, Pearson's chi-square test was used. The exact probability test was used for small-frequency distributions. The significance level was set at p<0.05.

## Results

Table 1 presents the sociodemographic characteristics of the 882 study participants from Saudi Arabia.

**Table 1:** Sociodemographic characteristics of the study participants (n=882) SAR: Saudi Arabian Riyal

Data	Number of participants	%
Region		
Central Region	381	43.2%
Northern Region	30	3.4%
Eastern Region	179	20.3%
Western Region	246	27.9%
Southern Region	46	5.2%
Age (in years)		
18-24	415	47.1%
25-34	101	11.5%
35-44	127	14.4%
45-54	135	15.3%
55+	104	11.8%
Gender		
Male	140	15.9%
Female	742	84.1%
Educational level		
Below secondary	30	3.4%
Secondary education	187	21.2%
Diploma	87	9.9%
Bachelor degree	495	56.1%
Post-graduate degree	83	9.4%
Monthly income (SAR)		
5000	477	54.1%
10000	126	14.3%
15000	151	17.1%
20000	69	7.8%
25000	59	6.7%
Marital status		
Single	474	53.7%
Married	363	41.2%
Divorced/widow	45	5.1%

Regarding regional distribution, the largest group was from the Central (n=381, 43.2%), followed by the Western (n=246, 27.9%), Eastern (n=179, 20.3%), Southern (n=46, 5.2%), and Northern (n=30, 3.4%) Regions. The age of the participants was predominantly between 18 and 24 years (n=415, 47.1%), with smaller proportions in the 25-34 (n=101, 11.5%), 35-44 (n=127, 14.4%), 45-54 (n=135, 15.3%), and 55+ (n=104, 11.8%) age groups. Female participants (n=742, 84.1%) were in majority compared to male participants (n=140, 15.9%). In terms of education, the most frequent level was a Bachelor's degree (n=495, 56.1%), followed by Secondary education (n=187, 21.2%), Diploma (n=87, 9.9%), Post-graduate degree (n=83, 9.4%), and Below secondary education (n=30, 3.4%). Concerning monthly income (in SARs), the largest segment earned 5000 (n=477, 54.1%), followed by 15000 (n=151, 17.1%), 10000 (n=126, 14.3%), 20000 (n=69, 7.8%), and 25000 (n=59, 6.7%). Finally, regarding marital status, the most frequent status was single (n=474, 53.7%), followed by married (n=363, 41.2%), and divorced/widowed (n=45, 5.1%).

Table 2 presents public perceptions regarding which medical professional (dermatologist, cosmetic surgeon) should perform various procedures.

**Table 2:** Public awareness and perceptions of procedures performed by dermatologists, cosmetic surgeons in Saudi Arabia (n=882) ^#^the correct answer

Procedure	Dermatologist		Cosmetic surgery		Both of them		I don't know	
	No	%	No	%	No	%	No	%
Skin cancer removal	410^#^	46.5%	84	9.5%	274	31.1%	114	12.9%
Cleft lip and cleft palate reconstruction	57	6.5%	558^#^	63.3%	202	22.9%	65	7.4%
Treating facial wounds	339	38.4%	226^#^	25.6%	290	32.9%	27	3.1%
Treating burns	420	47.6%	102	11.6%	331^#^	37.5%	29	3.3%
Congenital deformities of the ear and nose	63	7.1%	570^#^	64.6%	205	23.2%	44	5.0%
Botox injection	221	25.1%	488	55.3%	128^#^	14.5%	45	5.1%
Surgical facelift	73	8.3%	640^#^	72.6%	133	15.1%	36	4.1%
Facelift with threads	157^#^	17.8%	524	59.4%	149	16.9%	52	5.9%
Skin grafts	166	18.8%	406^#^	46.0%	245	27.8%	65	7.4%
Tattoo removal	313^#^	35.5%	304	34.5%	189	21.4%	76	8.6%
Remove Navi	300^#^	34.0%	296	33.6%	219	24.8%	67	7.6%
Treating acne scars	571^#^	64.7%	135	15.3%	146	16.6%	30	3.4%
Ingrown nail procedure	326	37.0%	227^#^	25.7%	151	17.1%	178	20.2%
Hair transplant	272^#^	30.8%	333	37.8%	192	21.8%	85	9.6%
Laser hair removal	497^#^	56.3%	222	25.2%	102	11.6%	61	6.9%
Treating surgical scars	242	27.4%	355^#^	40.2%	227	25.7%	58	6.6%

For skin cancer removal, the most frequent response was "dermatologist" (n=410, 46.5%). The vast majority correctly identified "cosmetic surgeon" (n=558, 63.3%) for cleft lip and cleft palate reconstruction. Regarding facial wound treatment, the most common perception was "dermatologist" (n=339, 38.4%), although a substantial portion also selected "both" (n=290, 32.9%). For treating burns, "dermatologist" was selected by 420 participants (47.6%), while a significant number (n=331, 37.5%) opted for "both." Congenital ear and nose deformities were predominantly and correctly associated with "cosmetic surgeons" (n=570, 64.6%). Botox injections were most frequently attributed to "cosmetic surgeons" (n=488, 55.3%), although "both" was also a common choice (n=128, 14.5%). Surgical facelifts were largely and correctly linked to "cosmetic surgeons" (n=640, 72.6%). Similarly, facelifts with threads were most often associated with "dermatologists" (n=157, 17.8%), although "cosmetic surgeons" had a higher selection rate (n=524, 59.4%). Skin grafts were primarily, and correctly, linked to "cosmetic surgeons" (n=406, 46.0%). Tattoo removal was most often attributed to "dermatologist" (n=313, 35.5%). "Removing Navi" (likely referring to nevi or moles) was most frequently associated with "dermatologist" (n=300, 34.0%). Treating acne scars was mostly incorrectly attributed to "dermatologists" (n=571, 64.7%). The ingrown nail procedure was most frequently linked to "cosmetic surgeon" (n=227, 25.7%), although "dermatologist" was selected by 326 participants (37%). Hair transplants were most often associated with "dermatologist" (n=272, 30.8%), although "cosmetic surgeon" was also a common choice (n=333, 37.8%). Laser hair removal was predominantly and correctly linked to a "dermatologist" (n=497, 56.3%). Finally, treating surgical scars was most frequently associated with "cosmetic surgeon" (n=355, 40.2%), although "dermatologist" was also a common selection (n=242, 27.4%).

**Figure 1:**
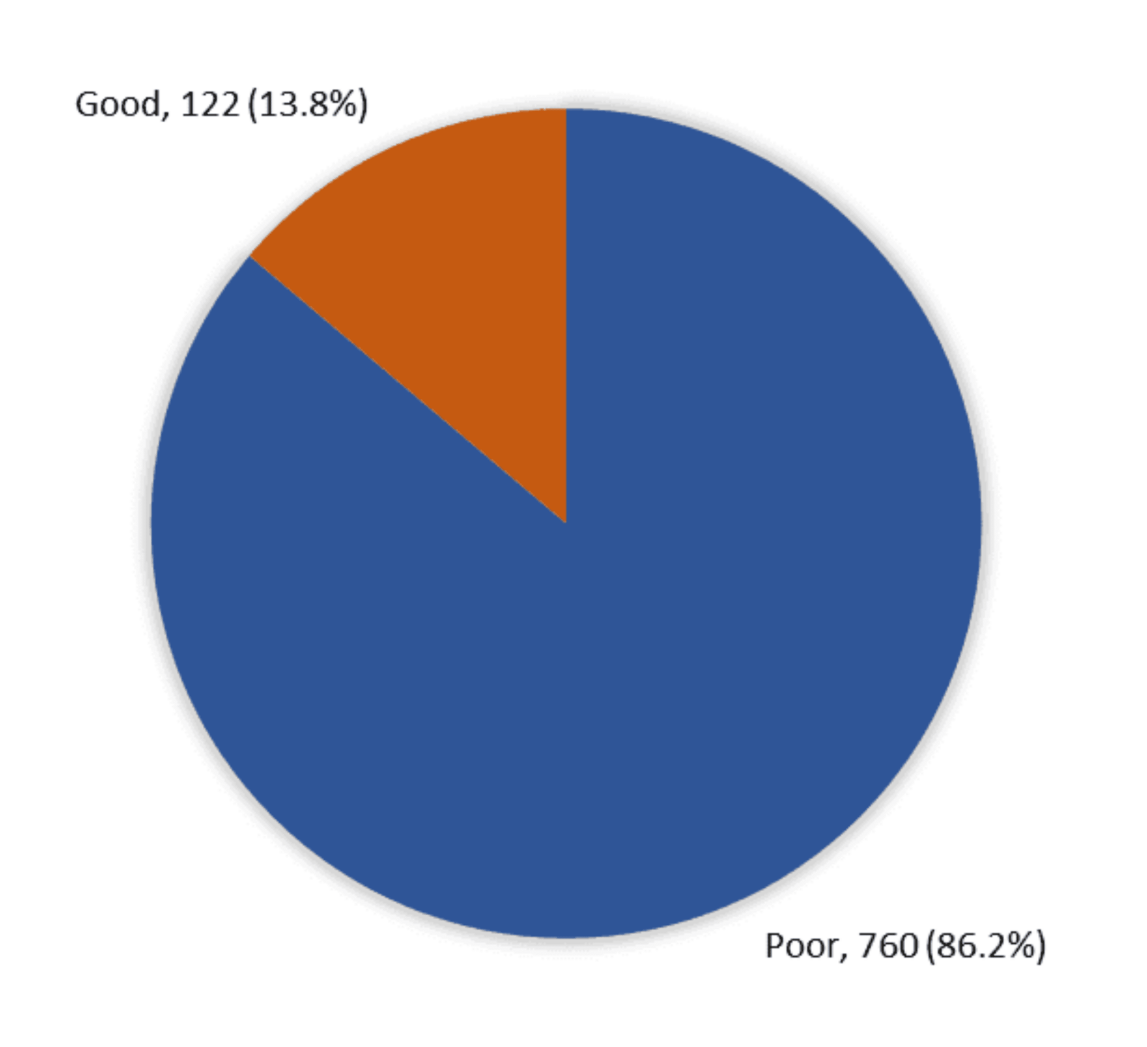
The overall public awareness and perceptions of procedures performed by dermatologists and cosmetic surgeons in Saudi Arabia (n=882)

Figure 1 displays the overall public awareness and perceptions in Saudi Arabia regarding procedures performed by dermatologists and cosmetic surgeons (n=882).

The data reveals that the majority of respondents (n=760), demonstrated poor overall awareness, representing 86.2% of the total sample. Conversely, only 122 participants exhibited good overall awareness, accounting for 13.8% of the total sample. 

**Figure 2:**
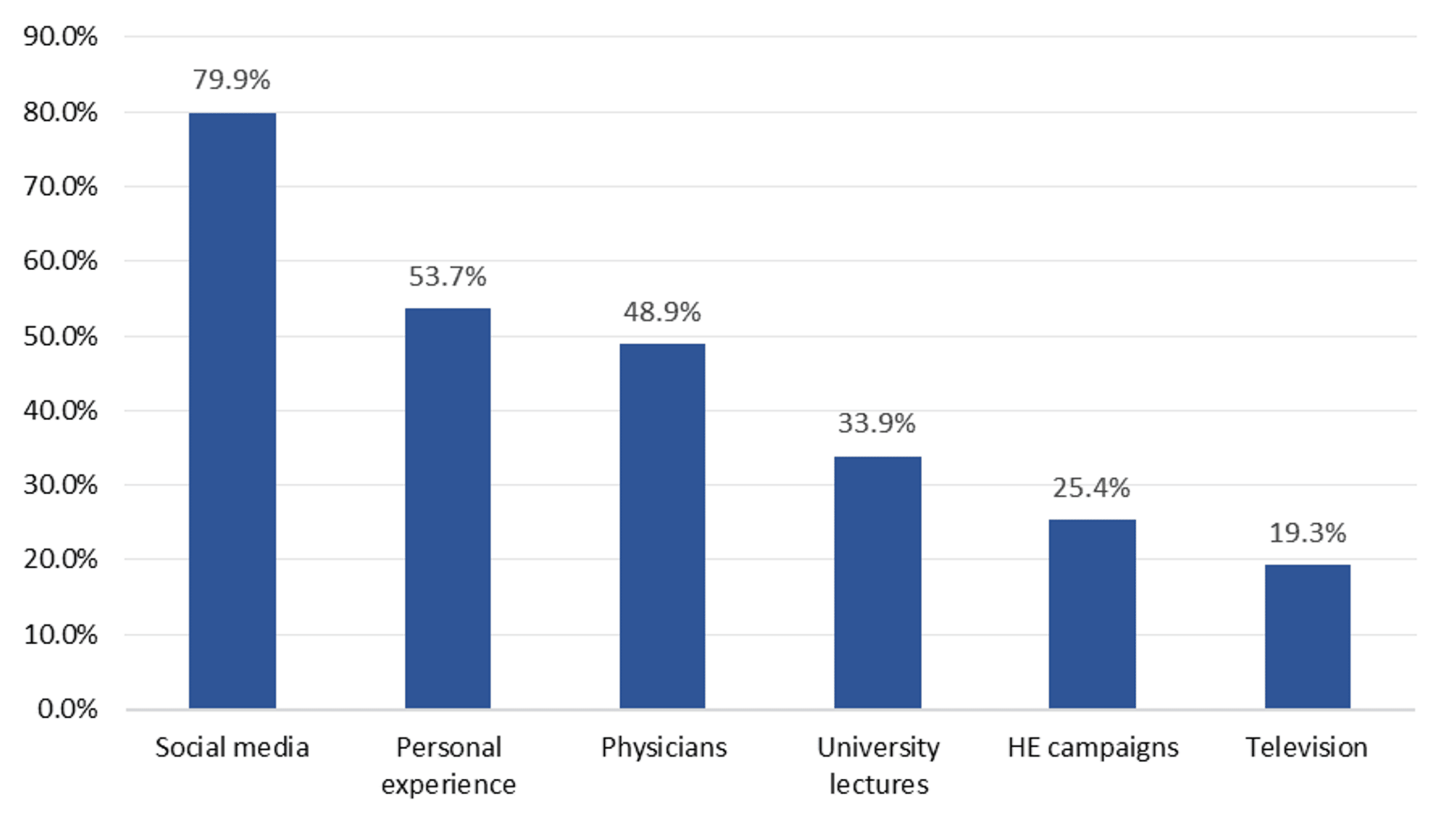
The source of information about the roles of dermatologists and cosmetic surgeons in Saudi Arabia (n=882)

Figure 2 shows the source of information about the role of dermatologists and cosmetic surgeons.

Social media was the most frequently reported source of information regarding the roles of dermatologists and cosmetic surgeons in Saudi Arabia, with 705 respondents (79.9%) reporting its use. Personal experience also played a substantial role, informing 474 individuals (53.7%). Physicians were reported by 431 participants (48.9%). University lectures were reported by 299 respondents (33.9%), while health education campaigns were reported by 224 participants (25.4%). Television was the least utilized source among those listed, with only 170 individuals (19.3%).

Table 3 assessed factors associated with public knowledge and awareness of procedures performed by dermatologists and cosmetic surgeons in Saudi Arabia.

**Table 3:** Factors associated with public knowledge and awareness of procedures performed by dermatologists and cosmetic surgeons in Saudi Arabia P: Pearson χ2 test; ^Exact probability test; *P<0.05 (significant)

Factors	Overall awareness level				p-value
	Poor		Good		
	No	%	No	%	
Region					0.105
Central Region	323	84.8%	58	15.2%	
Northern Region	28	93.3%	2	6.7%	
Eastern Region	159	88.8%	20	11.2%	
Western Region	206	83.7%	40	16.3%	
Southern Region	44	95.7%	2	4.3%	
Age in years					0.082
18-24	354	85.3%	61	14.7%	
25-34	80	79.2%	21	20.8%	
35-44	112	88.2%	15	11.8%	
45-54	118	87.4%	17	12.6%	
55+	96	92.3%	8	7.7%	
Gender					0.049*
Male	128	91.4%	12	8.6%	
Female	632	85.2%	110	14.8%	
Educational level					0.110
Below secondary	27	90.0%	3	10.0%	
Secondary education	159	85.0%	28	15.0%	
Diploma	83	95.4%	4	4.6%	
Bachelor degree	421	85.1%	74	14.9%	
Post-graduate degree	70	84.3%	13	15.7%	
Monthly income (SAR)					0.500
5000	408	85.5%	69	14.5%	
10000	114	90.5%	12	9.5%	
15000	126	83.4%	25	16.6%	
20000	61	88.4%	8	11.6%	
25000	51	86.4%	8	13.6%	
Marital status					0.023*
Single	395	83.3%	79	16.7%	
Married	323	89.0%	40	11.0%	
Divorced / widow	42	93.3%	3	6.7%	
Source of information					0.012*^
Social media	613	87.0%	92	13.0%	
Physicians	363	84.2%	68	15.8%	
Personal experience	411	86.7%	63	13.3%	
University lectures	252	84.3%	47	15.7%	
Television	159	93.5%	11	6.5%	
Health education campaigns	197	87.9%	27	12.1%	

While region, age, education level, and monthly income showed no statistically significant association with overall awareness level (p>0.05 for all), gender showed a significant association (p=0.049), with female participants having higher awareness (14.8%) compared to male respondents (8.6%). Marital status also showed a statistically significant association with awareness (p=0.023), with those who were single having a higher awareness (16.7%) compared to married (11%) or divorced/widowed individuals (6.7%). Finally, the source of information was significantly associated with awareness levels (p=0.012). Individuals who primarily used television as a source of information had the lowest rate of good awareness (6.5%), while those using social media, physicians, personal experience, university lectures, or health education campaigns as their information sources demonstrated higher rates of good awareness (13%, 15.8%, 13.3%, 15.7%, and 12.1%, respectively).

## Discussion

This study evaluated public awareness in Saudi Arabia regarding the respective roles of dermatologists and plastic surgeons in both medical and aesthetic procedures. A significant knowledge gap was identified, with 86.2% of respondents demonstrating poor awareness and only 13.8% showing adequate understanding of which specialist is appropriate for specific conditions. This lack of awareness may impact clinical decision-making, referral accuracy, and treatment outcomes.

Our findings are consistent with prior research conducted in the United States, which demonstrated that many members of the public fail to associate dermatologists with surgical procedures, despite their integral role in skin cancer treatment and various aesthetic interventions [9,10].

When examining specific procedures, 55.3% of our respondents selected plastic surgeons for Botox injections. In comparison, a 2017 study conducted in Saudi Arabia by AlHargan et al. reported that 87.2% of participants made the same selection [8]. Additionally, 29.2% of the study's participants selected dermatologists for Botox, whereas 35.5% did so in our study, suggesting a modest upward trend in public recognition of dermatologists’ involvement in such procedures. As an Arabic language native speaker, one factor that may have contributed to this confusion is the linguistic overlap between the terms "plastic surgeon" and "cosmetic surgeon" in the Arabic Language. Unlike English, where these designations are tied to distinct training and board certification pathways, Arabic often uses a single term to describe both. The lack of differentiation may have led some participants to confuse the roles. Recognizing this linguistic nuance is important for interpreting the result. 

Our analysis identified gender and marital status as statistically significant predictors of awareness. Female participants (14.8%) and single individuals (16.7%) demonstrated higher levels of awareness than their counterparts. These trends may be linked to greater aesthetic health-seeking behavior and increased exposure to cosmetic-related content among these demographic groups, as supported by prior research [11,12].

Social media was the most frequently cited source of information in our study, with 79.9% of participants citing it as their main source for dermatology and plastic surgery learning. This finding supports the growing influence of digital social media platforms on public health knowledge and aesthetic practice visibility. Previous literature has documented the role of social media in shaping perceptions, advertising services, and even driving demand for cosmetic procedures [13-16].

Interestingly, our data revealed no statistically significant correlation between awareness levels and education or income. This contradicts the conventional assumption that higher education and income correlate with greater health literacy, and suggests that awareness in this domain may depend more on media exposure and personal interests than formal education. No previous studies have explored this relationship in the context of dermatology and plastic surgery awareness, representing a novel avenue for future research.

Incorrectly attributing procedures to inappropriate specialists may result in care delays, increased healthcare costs, or suboptimal treatment outcomes. For instance, consulting a plastic surgeon for acne scars could lead to more invasive or expensive interventions when non-invasive dermatologic treatments would suffice. Conversely, referring congenital deformities like cleft palates to dermatologists could delay essential surgical care. These mismatches not only affect treatment efficiency but may also burden the healthcare system through misallocated resources [17-19].

Given the influence of social media and the apparent gaps in awareness among certain demographic groups, future public health campaigns should use social media platforms to deliver accurate, specialty-specific information. Moreover, research exploring physician referral patterns and public decision-making in real clinical contexts could provide deeper insights into the implications of public perception. Educational interventions such as infographic-based content, short-form videos, or clinic-based outreach could serve to reduce misperceptions.

There are some limitations to our study. First, the use of online, self-selected sampling via social media platforms introduces potential selection bias, resulting in overrepresentation of females and younger adults. This may affect the generalizability of the findings. Second, although the questionnaire was informed by previous literature, it was not formally pilot-tested or validated by tools like Cronbach's alpha. Lastly, the inability to track how many individuals viewed the survey link prevented the calculation of the response rate of the study. A limitation worth mentioning is the fact that Arabic language does not offer a linguistic distinction between a "plastic surgeon" and a "cosmetic surgeon." 

## Conclusions

In conclusion, this study aimed to investigate the level of awareness and understanding of the roles of dermatologists and plastic surgeons among adults living in Saudi Arabia. The findings indicate that the majority of respondents demonstrated poor overall awareness regarding dermatological and cosmetic procedures. Addressing the awareness level and understanding the roles of dermatologists and plastic surgeons among adults living in Saudi Arabia is a critical step towards improving the health and well-being of the general population.
